# Observations on Those Diseases of Females Which Are Attended by Discharges

**Published:** 1822-03-01

**Authors:** 


*Observations on those Diseases of Females which are attended by Discharges. Illustrated by Copper-plates of the Diseases.* By Charles Mansfield Clarke, Member of the Royal College of Surgeons, London. Part II. Octavo, pp. 243. Four Plates. Longman, London, 1821.

"Helleborum frustra, cum jam Cutis Œgra tumebat,
"Poscentes videas;—venienti occurrite morbo."—*Pers. Sat.* 3.

This work was long promised by the author, and anxiously expected by the profession. The majority of medical works, now-a-days, are written to *procure*, not to *record*, practice, like a surgeon, some 20 years ago, who advertised a book *in the press*, on a particular operation, in order thereby, to get some cases to compose the work, not really commenced in manuscript! When, therefore, a practitioner, of Mr. Clarke's standing and experience, condescends to lay open the results of his observations to the public at large, we naturally attach a comparatively high degree of importance to the publication, as one that will not be likely to lead us astray, though it may fall short of the expectation which we too sanguinely indulge, respecting the productions of men standing high in professional rank and reputation. The name of Clarke, too, has long been associated with obstetric studies and pursuits, the gifted brother of the present author having left an indelible impression on the minds of all his numerous pupils. Alas poor Yorick! He, whose wit so often set the class-room in a roar, is now, in his turn, chap-fallen and silent! A few years more, and those who still cherish his memory, will also sink in the tide of time, never to rise again! But it is useless to repine. The whole frame of Nature and all its minutest parts are undergoing the same revolutionary process, and eternally changing forms, by an irresistible action and re-action of their own constituent elements. The sea sends forth its rains, that soften, and wash, and wear down the hardest rocks and highest mountains; while these debris in time accumulate, and encroach on the dominions of the ocean. Changes and contentions of this kind, are going on in every thing, from the continents and seas of the earth, to the solids and fluids of an animalcula. Man, with all his towering intellect and proud prerogatives, falls into the mysterious circle of transmutation, and nothing is permanent in this world, but the laws which govern its instability!

The first volume of Mr. Clarke's work has now been long before the public, and its contents well known. In the present volume, our author has entered into the consideration of many diseases which, he thinks, have hitherto been but imperfectly discriminated from one another, and treated with little regard to any principles. Mr. Clarke justly observes that, although many of these diseases are invariably fatal, yet the acceleration or retardation of their progress depends upon the knowledge of the practitioner. Many of them, too, are accompanied with great pain—and, whether that pain is to be aggravated or subdued—whether the patient is to be allowed to expire in torture, or slide into eternity with mitigated suffering, will be determined by the information of the medical attendant, and his acquaintance with the means of diminishing those physical ills to which humanity is subject. These are cogent reasons why the medical practitioner should allow no opportunity of augmenting his knowledge to pass unimproved. Neither should he proudly and obstinately rely on his own observations alone, but listen meekly to the suggestions and remarks of others also; for it has been wisely said, that as "the greatest friend of Truth is Time, so her greatest enemy is *Prejudice*, and her constant companion, *Humility*."

Mr. Clarke has comprehended all the discharges from the vagina under the following five heads, *viz.* Transparent Mucous Discharge—White Mucous Discharge—Watery Discharge—Purulent Discharge—and Sanguineous Discharge.* The *first*, or Transparent Mucous Discharge, has been treated of in the preceding volume, and to that we must refer. Our next subject, therefore, is the—

I. *White Mucous Discharge*. Our author has defined this to be opaque, and perfectly white in colour, resembling a mixture of starch and water, or thin cream, easily washed from the finger, and capable of diffusion in water, rendering the latter turbid.—This discharge appertains to but one morbid state of the uterus, but characterizes that state very constantly. It probably results from a morbid state of the glands of the cervix uteri, for when pressure is made upon that part, the woman complains of considerable pain. The examination of this discharge should be made when the patient has been some time quiet, for the *transparent* mucus, if

* Mr. Clarke very properly excludes from his classification, gonorrhœa, for what could be more absurd, than to term that a "disease of *females*," which is equally common to both sexes?

so abundant as to run over the labia (where there is some degree of friction in walking) becomes also opaque and white —probably from the entanglement of air globules with mucus. Such a mixture, however, of air and mucus, will *not* render water turbid. The white mucous discharge is often thicker than cream, having the consistence of glue, corresponding with the mucus separated from the cervix uteri, at the commencement of labour. When possessed of this degree of tenacity, it does not flow spontaneously, but generally when there is some straining at stool. It may, however, by remaining in the vagina, become mixed and attenuated with the mucus of that part.

It is at all times desirable to ascertain the real nature of a vaginal discharge, for the white mucus in question is often mistaken for pus, and thus the patient and practitioner are harrassed with the dread of a formidable disease. Women are most liable to the white opake discharge between the age of 20 and 45. Mr. C. thinks it not unlikely, though he speaks cautiously, that this secretion from the glands of the cervix uteri, may be a forerunner of some of those important diseases of that part, as carcinoma for instance.

"The constitution is rarely affected in this complaint, the action of the heart and arteries is not increased, and the functions of health are seldom interrupted.

"The menstruation is seldom affected, but it proceeds as it was accustomed to do in such persons. In some instances, painful menstruation has been present. Where an examination per vaginam has been made, the external parts and the canal of the vagina have not possessed a more than ordinary degree of sensibility, but upon the finger reaching the cervix uteri the patient has complained of pain, and the uneasiness has been compared to that which has been experienced upon the passage of an evacuation from the rectum; pressure in both cases being the cause of the pain. There is, however, no alteration of structure in the part; no thickening, no peculiar enlargement of the os uteri, no breach of surface; the portion of the vagina which is reflected over the cervix uteri possessing its usual polished and smooth state." P. 11.

Our author is now confident that wherever the white mucous discharge is present, there will be found, on examination, a tenderness of the cervix uteri, which will be removed, or relieved, by the mode of treatment hereafter to be described. Some cases, however, resist all treatment, and at length wear themselves out—in many instances preventing impregnation during their continuance. Here our author relates a case, which we shall give in his own words.

"A lady, about twenty-five years of age, who had been married two or three years, but who had never fallen with child, complained of a considerable degree of uneasiness at the extremity of the back, near the os coccygis; on this account she indulged much in the horizontal posture. She had also been liable for some years to a discharge from the vagina, which, on investigation, was ascertained to put on a white appearance: the general health was tolerably good, excepting that at the periods of menstruation great pain was felt at the bottom of the belly, which lasted for twenty-four hours, during which time the menstruous discharge did not flow freely, but was pale, and occasionally mixed with portions of a stringy substance. On account of the discharge, astringent injections had been employed by a practitioner, who had been consulted, but without any effect upon the complaint or its symptoms. Tonics had also been exhibited without any advantage. An examination being allowed, the uterus was found unusually low, and the neck of it possessed a much greater degree of sensibility than is common; so that, pressure being made upon it, the patient complained much; but this increased sensibility did not extend to the neighbouring parts, neither was there any alteration in the structure of the parts. This lady had been in the habit of taking much riding exercise, and it is more than probable, that to this cause was to be attributed both the tenderness of the cervix uteri, and the descent of the whole organ. Prolapsus uteri being a very infrequent disease in women, who have not borne children, the patient was desired to lose several ounces of blood from the loins, to live temperately, to avoid riding exercise, to take only a sufficient quantity of walking exercise to keep herself in health, and to inject some tepid water into the vagina. Sexual intercourse was of course interdicted. Soon after this plan was instituted the symptoms diminished. On account of the painful menstruation, some diaphoretic medicines, with opium, and the use of the hip bath, were recommended, and the sufferings at the periods were subdued. At the end of three or four months, the complaints were removed; but the patient did not become pregnant." P. 13.

In some cases the excitement is propagated from the cervix uteri to the neighbouring parts. A young woman, 22 years of age, irregular in life and habits, had borne a child at 18. She afterwards became gradually affected with pain at the bottom of the abdomen, extending towards the back, and from which, she was never wholly free, though it was increased by sexual intercourse. A milky discharge accompanied these symptoms.

"On making an examination, the entrance of the finger was impeded by an encysted tumour, containing a fluid upon the right side of the vestibulum, and extending upwards towards the vagina. The tumour was as large as a pigeon's egg, and was insensible: on carrying the finger towards the uterus, the neck of this organ was found exceedingly tender upon pressure." P. 16.

Our author being desirous of ascertaining how far the loss of blood, attending a removal of the tumour might prove aremedy for the inflamed cervix uteri, made an incision into the parts, and, dissected out the tumour. The hæmorrhage was excessive, but was stopped by plugging the vagina. The wound granulated and the sore healed. The discharge stopped, and the uterine tenderness ceased. Six years afterwards, our author and Dr. Maton were consulted on the case of the same female. The pressing symptom now, was a distention of the bladder, without the power of expelling its contents. The urine was drawn off by the catheter, and on carrying the finger into the vagina, the uterus was found enlarged to the size of an orange, and having fallen backwards into the hollow of the sacrum, its cervix made pressure on the meatus urinarius. In consequence of continued intemperance, chronic enlargement of the liver had also taken place, the skin of the patient having assumed a dirty appearance. Profuse menstruation had come on, and increased the debility. The termination of such a combination of symptoms may be easily prognosticated.

"In the preceding history may be traced the progress of a disease at first shown only by increased action of vessels, which was removed by blood-letting and by rest, for a time: but the flame, though subdued, was not extinguished:—ready to be lighted up, upon the application of those exciting causes, debauchery and intemperance, it was rekindled; and, in all probability, at a period not very distant, will consume the frame which engendered it." P. 18.

Encysted tumours are not unfrequently met with in the neighbourhood of parts where increased action is going on, as in the labia, and in the cellular membrane about the vagina, when local inflammation has existed in the vicinity. A case in illustration, which had been under Mr. Freeman, of Spring Gardens, is related at page 19, which was shortly this. A young healthy woman being married nine months, found her health begin to decline, and the intervals of menstruation to be protracted. Sexual intercourse was painful, and there was a discharge of whitish mucus from the vagina, at the upper part of which, there was constant uneasiness. On examination, a tumour, the size of a walnut, was found on the left side of the vagina, and was lowered by the patient's straining. The cervix uteri was painful when touched. The constant irritation of the parts, kept up general increase of the circulation, and this produced some degree of emaciation. As the tumour evidently contained a fluid, it was punctured with a lancet, and a clear liquid escaped. Treatment for diminishing the local increased action was pursued, and perfect rest was enjoined. In a few days, the tenderness of the cervix uteri was nearly gone—the milky discharge ceased, and the patient shortly regained her health.

Our author gives a case to shew that the constitution sometimes sympathizes, though not very often, with the local irritation. A married lady had, for some time, been suffering from pain at the bottom of the back and abdomen, which continued to increase, for a few days, at the end of which, a violent paroxysm of fever took place, which was repeated in some hours afterwards. Twelve ounces of blood were taken and exhibited the inflammatory appearances. Salts and senna were exhibited, and on the surface of each evacuation, the patient observed a quantity of substance, resembling a solution of isinglass. This was discovered not to come from the rectum, but was squeezed out of the vagina, after the fæces escaped from the anus. When the bowels were evacuated, small doses of antimony and mercury were given, and the patient kept to her room on abstemious diet. The pain now abated, the glutinous discharge from the vagina ceased, and the excitement of the system subsided.

The above cases will be sufficient to illustrate the subject. We shall now exhibit a sketch of our author's therapeutical indications in a general way.

Local blood-letting, by cupping or leeching the back or groins, repeated according to circumstances, forms the basis of the treatment; and where symptomatic fever presents itself, venesection is proper, though seldom necessary. The hip-bath is a useful remedy, and the patient may sit in it twice a day, at the temperature of 90°. Where this last cannot be procured, fomentations to the back or abdomen, are serviceable. Tepid water thrown into the vagina with a syringe, constitutes a direct fomentation to the part affected. The bowels are to be kept in a relaxed state by small doses of magnesia, in plethoric habits;—in languid constitutions, castor oil may be preferable. No drastic or irritating purgatives should be given. At bed-time, it is proper to exhibit some medicine that may determine to the surface, and, at the same time, tranquillize the system. Thus, five grains of Dover's powder, and three of camphor, may be given with a saline draught. If strangury be considerable in degree, a larger dose of opium will be necessary, such as 60 or 80 drops of laudanum, and smaller doses frequently repeated afterwards, with mucilaginous drinks. Whenever the bladder is unequal to the expulsion of its contents, the catheter should be used, lest inflammation be superadded to irritation. We should never trust to diuretics, as nitric ether, and steaming with warm water, in these cases. Although it is not often necessary to keep the patient in bed, yet the horizontal position should be persisted in for some time, and all new causes of irritation avoided.

The disease which most nearly simulates the above is—

*Inflammation of the Unimpregnated Uterus.* In this, besides the pain arising from local inflammation, which, of course, is *permanent*, there will be occasional pains, which come on and retire after the manner of early labour pains. Besides, a milky discharge from the vagina does *not* accompany hysteritis, in which complaint pressure above the pubis greatly aggravates the pain.

Hysteritis is not an uncommon disease Mr. C. thinks; nor is it attended with symptoms so acute as might be expected when the unyielding texture of the uterine muscular fibres is considered. It must be recollected, however, that the uterus is not a very sensible part (with the exception of its cervix) and that it is well defended from pressure by the pelvic circle of bones. In the *impregnated* state these circumstances are all very different—there is then a greater extent of inflammation—the nerves and blood-vessels of the organ become enlarged—and the part is constantly under the influence of pressure from the abdominal and diaphragmatic muscles. Hysteritis in the unimpregnated state is most frequently called into action by local violence—and is not an uncommon consequence of marriage. Cold also may originate it. It is attended by a constant uneasiness referred to the pelvis, which gradually increasing, though seldom intensely violent, yet greatly interferes with the comfort of the patient, who complains of pain at the bottom of the abdomen or back. Besides the permanent pain, there are occasionally violent paroxysms of it, at irregular intervals—a phenomenon common to inflammation of most muscular organs, as the stomach, bladder, &c. In the unimpregnated hysteritis the circulation is seldom much accelerated, nor is there much hardness in the pulse, or increased temperature of skin. The tongue, though not clean, does not shew that slimy whiteness so constant an attendant on peritonæal inflammation. The disease lasts a long time if not interfered with, the symptoms suffering an exacerbation before each menstrual period, and a diminution afterwards. In some cases the menstruation is suspended, and the symptoms aggravated. In inflammation of the substance of the uterus, and where the peritonæal covering has not participated, the blood drawn from a vein seldom shews the inflammatory crust, nor is the relief commensurate with the activity of the means, or answerable to the expectations of the employer. Topical blood-letting is more advantageous. Scarifications and cupping glasses to the sacrum—a dozen of leeches to each groin, or across the pubis—and immersion of the hips in warm water, are the appropriate measures. At the end of a week, the local bleeding should be repeated, and in the intervals of menstruation a dozen of leeches to the neighbourhood. Twice or thrice in the 24 hours a fomentation, composed of decoct. anthemidis and tincture of opium, an ounce of the former to a quart of the latter, should be applied, and if the symptoms do not seem disposed to yield, the patient should be kept in bed, and small doses of antimony in a saline draught exhibited once in four or six hours, with three or four drops of tinctura opii. Purging is eminently useful in allaying inflammatory action, and therefore should not be neglected. Salts and senna every second morning are recommended by our author. The diet, of course, should be light and unirritating.

The general state of the health should be attended to, and the regularity of the menstrual secretion. We believe, with our author, that much mischief is daily done by the routine practice of adminstering what are termed emmenagogues in all states and conditions of menstrual irregularity. Most of these emmenagogues are either general or local stimulants, and consequently cannot be proper in the various conditions of obstructed menstruation. Well may our author observe that "prejudice has occupied the place of science, and popular nostrums have been exhibited, often without, and sometimes with, the concurrence of the practitioner." Every attentive observer knows that, if there be cases of obstructed or suppressed catameniæ, where the fluid is tardily secreted from local or general debility, there are many other cases in which an opposite state of the frame becomes the cause of their production.

"Instead then of resorting to such measures, to the employment of the whip and of the spur in such cases, (where if they do any thing, they do mischief,) let the morbid peculiarities of the constitution, and the habits of life of the patient be taken into consideration; let the first be counteracted, the second be improved; let the sanguine have her excess of fulness diminished, let the debilitated have her powers augmented; in short, let the general health be amended, and the functions of health will be restored." 39.

Accidental circumstances, as the application of cold or fatigue, may have interrupted the menstrual discharge by exciting fever in the system. In this case confine the patient to bed—supply her with cool drinks—open her bowels—and, if medicine must be given, pro forma, exhibit the saline draughts. Under this treatment the feverishness subsides, and with the restoration of health there will be a restoration of the uterine functions.

Luxurious living often induces amenorrhœa by deranging the balance between the wear and tear of the system, and the supply of food, thus producing a plethoric and unhealthy state of the constitution. Here abstemiousness with increase of exercise is the remedy. And should it be inefficient of itself, venesection (full bleedings rather than repeated small ones) with saline purgatives, must be resorted to.

"The patient should not lose less than from sixteen to twenty ounces of blood at once. Under ordinary circumstances it is very immaterial from what part of the body the blood is taken, provided the vessel is large and the orifice in the vein sufficiently so to allow the blood to escape rapidly; but if there should be any evidence of local congestion, it will be right to remove the blood from the neighbourhood of that part, as from the external jugular vein when there is pain in the head and giddiness." 43.

Lastly, should confinement, breathing an impure air, or poverty of living, have injured the general health, inducing debility and obstruction, and constituting chlorosis, then it will be necessary to invigorate the frame by every means in our power. The stomach must be strengthened, first by the lighter bitters and most digestible food, proceeding gradually to the use of tonics more powerful, as bark and steel. It is only when the constitutional weakness has been removed by such means, that we should have recourse to stimulants. Our author thinks it improbable that there are any medicines which exert a specific effect upon the uterus. In this we cannot agree with Mr. Clarke. There are very few organs in the body which are not peculiarly acted on by certain substances taken into the stomach: thus lytta stimulates the kidney, mercury the liver—and we may pretty securely affirm that the *secale cornutum* acts specifically on the uterus. However this may be, the volatile alkalis, spices, essential oils, and wines are useful general stimulants in the chlorotic state abovementioned; but the cold bath is a precarious remedy, especially when the stomach is unusually weak. The sabina, lytta, black hellebore, many of the resinous gums, electricity, and horse exercise, are stimulants which Mr. Clarke considers as exerting their influence "upon parts in the vicinity of the uterus." One drachm of the tinctura sabinæ composita may be given twice a day in some aromatic bitter draught, and the tincture may be increased to two drachms. Lytta, he thinks, irritates the urinary passages; and, through that medium, the uterine system, a knowledge of which circumstance is available in the case of obstructed menstruation. A blister, therefore, may be applied to the sacrum; or ten drops of the tincture may be taken internally at first, and increased to thirty, in infusion of cascarilla, or any convenient vehicle. As a substitute for lytta, a drachm of the tincture of black hellebore may be taken. Thus, galbanum and aloës possess the power (especially aloes) of stimulating the rectum, and unless given so as to produce this effect, they are of little service in amenorrhœa. They are preferable to the oily or saline purgatives. When excessive irritation about the anus exists, the infusum lini may be injected (℥j.) once or twice a day, the other medicines intermitted for a few days.

In cases of dyspepsia, *unaccompanied by organic disease of the viscera,* gestation, particularly riding, is useful; "not so when organic changes are suspected to exist; as these have inflammation for their basis, it is evident that whatever excites action in such parts, will augment the mischief." We think this observation requires some modification. We believe that in chronic inflammation of the serous membranes and parenchymatous structure of organs, the succussions of horse, or even carriage exercise, are injurious. But in chronic inflammations of the *mucous* membranes, as of the lungs and bowels, horse and carriage exercise is peculiarly beneficial—a remark as old as Celsus, and confirmed by modern experience. "Gestatio *longis* et jam inclinatis morbis aptissima est, &c."—*Cels.* Dr. Currie, of Liverpool, when threatened with phthisis, found that horse exercise invariably equallized the balance of the circulation, and rendered the pulse slower. Hence in those cases of irritability of the heart, accompanied by palpitation or other irregular action, gestation on horseback, or in a carriage, is peculiarly advantageous. With these exceptions and limitations, we agree in the general correctness of our author's precept.

Mr. Clarke makes the same objections, and properly, to tonics and stimulating purgatives, when actual organic disease of the uterus, or indeed any viscus, exists.

> "Under a treatment of a mild character, the occasional application of a few leeches, the administration of a little manna, oil, or magnesia, and small doses of hemlock, and under a diet at once soft and nutritious, but by no means stimulating, the author has known several instances of patients living many years, even when emaciation had taken place to a great degree; when, after death, disease (the result of slow inflammatory action,) was discovered, and that to a considerable extent, in the pylorus, the small intestines, and the liver." P. 49.

The remaining emmenagogue is electricity, the powers of which are very considerable in exciting the uterus to vigorous action. By means of it, says Dr. Clarke, "a great number of cases of amenorrhœa have been cured, when no other means had been successful." Like many other emmenagogue remedies, however, it can never be useful till the powers of the system have been restored, and until the general health has been established.*

But to return from this digression to the subject of inflammation of the cervix uteri. Our author observes, that these cases often occur in habits where there is much inequality in the distribution of the blood, and consequent debility—resembling those cases where the glands of the neck, axilla, groins, or mesentery are in a state of chronic inflammation. These cases, we know, do not admit of antiphlogistic treatment. The cervix uteri is clearly a glandular structure, and Mr. C. thinks that it will be particularly liable to take on disease in habits which are prone to other glandular complaints. In phlogosis of the cervix uteri, therefore, in such habits, we must endeavour to equalize the balance of the circulation—"an object frequently attainable by the exhibition of tonics, amongst which the Peruvian bark and some preparations of iron, are the most serviceable." The decoct. cinchonæ, and tinct. muriat. ferri are the preparations recommended. Our author confesses, and with reason, that when the balance of the circulation inclines to any particular organ, as the head, the chest, the uterus, &c. it is no easy matter to conquer the disposition. It is no wonder that it should be difficult, because the disorder lies in the vessels of the organ itself, and not in the general circulation; for the heart can have no power of unequally distributing the blood. All irregular determinations depend upon the part where the congestion or plethora takes place.

In conclusion, our author thinks it a great point gained, if we can ascertain the true nature of this disease, of which the milky discharge is symptomatic, for thereby the practitioner is led to direct such measures as may tend to remove its cause, instead of those astringents too often employed on such occasions. It should be recollected that the cervix uteri is the seat of the disease—that this is the most sensible part of the uterus—that it is the part which carcinoma attacks—and that it is highly probable that slow inflammation there may lay the foundation for incipient carcinoma or other organic change. On all these accounts we cannot be too careful to remove so important a morbid process as chronic inflammation.

II. *Chap.* II. *Watery Discharge.* A discharge resembling clear water, containing very little or no glutinous

---

* For a paper on the effect of electricity in amenorrhœa by Dr. Austin, see the third volume of "Essays Physical and Literary." Ed. 1760.

matter. This results from cauliflower excrescence of the os uteri, hydatids of the uterus, or oozing excrescence of the labia. We shall notice them in their order.

*Cauliflower Excrescence.* Mr. Clarke prefaces the consideration of this subject by some remarks on the great tendency to disease in the organ which forms man's first nidus. Its outer or serous membrane is liable to peritoneal diseases —its muscular structure to irregular actions and tumours— its inner or secreting surface "is more liable to attacks of inflammation than any mucous membrane in the body"—its cervix or glandular structure to the diseases of glands in other parts—whilst the termination of this part in the vagina, at the os uteri, where it is covered by the inner membrane of the vaginal canal, is disposed to take on different forms of disease, one of which forms is that which stands at the head of this chapter.

"A more appropriate name could not have been given to this disease, than 'the cauliflower-excrescence.' There is a striking resemblance between itself and a portion of the upper surface of a cauliflower, or a head of brocoli. The surface is granulated, and it consists of a great number of small projections, which may be picked off from the surface, as the granules may be detached from the vegetable. The firmness of the tumour agrees also with that of the plant —here the granules will be large and irregular, there small and equal." P. 59.

From a very fine membrane spread over the surface of this tumour is poured out the aqueous secretion. As the tumour occupies the upper part of the vagina, it is concealed from view; but in some cases where it attained a considerable size, our author saw it, and it had a bright flesh colour. It pours out arterial-looking blood very plentifully, if injured during examination, and sometimes spontaneously, in plethoric habits. This excrescence has little or no sensibility—can never be traced into the cavity of the uterus—is sometimes rapid in its growth, but much influenced by the contractile power of the vagina, being more rapidly developed in married women who have borne children, than in single women. Our author has not been able to trace any cause of the disease, nor any predisposition connected with age or other circumstance, excepting he never met with it in women under twenty years of age.

"Perhaps some small arteries near the os uteri may undergo that morbid dilatation of their coats which is analogous to aneurism in larger trunks, and thus the disease may be produced. Something similar to this takes place in the arterial, or blood-red nævus, but here the surface, being covered by cutis and cuticle, no moisture of the part is met with; but if the surface of such a nævus should be injured, arterial blood escapes." 63.

No preparation of the cauliflower excrescence is preserved in any museum to which our author has had access, and he thinks the reason is, that the tumour almost wholly disappears after death, on account of its great vascularity. This was the case in two or three instances that fell within Mr. Clarke's own observation, where the tumour was perfectly distinct during life, but where, on dissection, nothing but a soft, whitish, flaccid substance, hung down from the os uteri resembling the fœtal portion of the placenta of a calf after maceration in water. We shall here abbreviate an interesting case related by Mr. Clarke, and where the practical indications were very puzzling.

Margaret Pole found herself pregnant of her ninth child in the beginning of 1810, and had a profuse watery discharge from the vagina, during the whole term of utero-gestation, sometimes tinged with blood, on any trifling exertion. In July the attendant practitioner found her in labour, and, on examination, felt a large tumour, resembling placenta, in the vagina, with a considerable discharge of blood. Mr. Clarke was then called in. In addition to the hæmorrhage there was constant vomiting, and consequently great exhaustion. The pulse was feeble and frequent, occasionally lost till the action of vomiting roused the circulation.* Mr. Clarke, on examination, found the vagina nearly filled with a substance resembling placenta; but on tracing it upwards he ascertained that, instead of coming down through the os uteri, as in placental presentations, it actually constituted a portion of the os uteri, so that there was scarcely any part of the circumference of this opening to which the tumour was not attached. As the os uteri was very little dilated, it was agreed that light nourishment and the usual remedies for checking uterine hæmorrhage should be exhibited. In a few

---

* We have had several opportunities of observing the great difference in effect between nausea and vomiting. If a vein happen to be open, no blood will be got during nausea; but as soon as actual vomiting occurs, the blood will spring forth with astonishing force, and completely resemble arterial blood in brightness of colour. From this we see that the action of vomiting, by driving the blood to the surface of the body, (for we observe the whole of the cutaneous vessels gorged during vomiting) frequently relieves internal congestion, or even hæmorrhage, as we have had some opportunities of witnessing. This may explain the reason why emetics have been recommended by practitioners in hæmoptysis and other internal bleedings, and perhaps may shew that there is less danger from vomiting even in apoplexy, than is generally imagined. *Rev.*

hours the os uteri opened more, the uterus began to act, and a profuse discharge of watery fluid, tinged with blood, came forth.

"Under this lamentable combination of circumstances, the existence of a formidable and fatal disease, and the presence of labour, the great question was, how the patient should be treated. The head was too low in the pelvis to admit of the child being turned; to open the head would have been to destroy the child, supposing it to be still alive, to afford no advantage to the woman; to perform the Cesarean operation, would have caused, in the deplorably weak state of the mother, her immediate death, and that when it was doubtful whether the child was alive or not." 69.

The tumour being evidently of the cauliflower species, our author naturally concluded that it would become diminished in bulk by the pressure of the child's head. It was therefore determined to wait and to watch the progress of the labour, apprising the friends of the perilous situation of the patient. The labour terminated naturally, but the woman sunk on the third day after delivery.

"The body was examined; and upon cutting open the vagina, the tumour had wholly disappeared, there remaining in its stead loose irregularly-shaped flocculent portions of matter, which arose from every part of the circle of the os uteri. There was nothing else found remarkable; and the uterus was as much contracted as it is usually found to be about three days after delivery." P. 70.

As the tumour under consideration is liable to be mistaken for placenta, it may be remarked, en passant, that a carcinomatous thickening of the os uteri bears no resemblance to the cauliflower-excrescence, its size remains unaltered by pressure, and undimininished by death. The cauliflower-excrescence and placenta, in fact, differ only (Mr. Clarke says) in name—the structure is the same.

*Symptomatology*. A perception of moisture first, and then gradually an inconvenient discharge, for which perhaps more abundant lavation with cold water is used, or the family receipt—isinglass and milk, internally. Still the discharge increases, but being unattended by pain or fœtor, the complaint is neglected, until a tinge of blood is perceived, or the cheek begins to change the rose for the lily, with a corresponding loss of strength. Then the alarm is taken. A discharge of blood almost always succeeds sexual intercourse—the digestion begins to be impaired—hysterical symptoms are produced, and all that host of inexplicable phenomena consequent on derangement of the chylopoetic viscera, increasing the patient's stock of bodily and mental misery.

Increase of debility is accompanied with decrease of absorption, and, of course, with depositions of fluid in different parts of the body, producing œdema of the feet at night, and puffiness of the face and eyelids in the morning. On this account, hydrothorax may destroy the patient long before she would have been exhausted by the disease itself. An alarming hæmorrhage sometimes induces a fatal syncope. "In many cases (says our author) the practitioner overlooks the disease, contenting himself with treating symptoms, without thinking of their cause." No great degree of emaciation, in general, attends the complaint. On the contrary, our author found, in several dissections, a layer of fat, of considerable thickness, covering the abdominal muscles. How different, (says Mr. Clarke) is this from the case of a patient destroyed by ulcerated carcinoma of the uterus, in which it is as easy to see the form and processes of the bones, as in a skeleton.

"Yet, as in this complaint a discharge is present, as now and then it is fœtid, as a tumour is found upon examination, and as the disease has always, sooner or later, a fatal tendency, it has been too frequently confounded with carcinoma. The prognostic, as to the *ultimate event*, it is true, must be the same; but the terms *sooner* or *later* admit of considerable latitude, and it is a great comfort to be enabled to lengthen life under such circumstances. It is not here, as in carcinoma, that whilst life is lengthened by art, distress and suffering is eked out with it. A patient labouring under the cauliflower excrescence may pass, nay, she may enjoy, several years of life, if she will be content to make some sacrifices." 87.

The discharge, and indeed the danger, being in proportion to the extent of surface of the tumour, it follows, that loss of tone in the vagina, is an unfavourable sign, as the pressure of a contracted vagina checks the development of the tumour. The danger is less also, when the tumour occupies only a small portion of the os uteri, than when the whole circumference of the opening is involved in the disease.

*Therapeia.* The enlargement of the tumour will be greatly retarded—nay, there is reason to believe that the size of the tumour will be *diminished* by judicious management—particularly by diminishing the action and fulness of the bloodvessels of the neighbouring parts. Mr. Clarke thinks that, by a steady perseverance in the proper measures, especially if taken early, "the farther progress of the disease may be put a stop to."

Local blood-letting from the region of the sacrum and hips, is a most valuable remedy. The quantity of blood to be taken away, must be regulated by the size and degree of resistance in the tumour, and by the quantity of watery discharge (always a measure of the extent of disease) regard being paid to the strength of the patient.

"At the same time it must be recollected, that if, by the loss of eight or ten ounces of blood by cupping, the quantity of the watery discharge can be diminished from four ounces to two ounces daily, the patient will, at the end of a fortnight, possess more power than if she had lost four ounces of blood by cupping, and the quantity of the watery discharge had been diminished to three ounces daily." 91.

Local bleeding, however, when intemperately employed, may hasten the patient's dissolution. It should not be prescribed when much œdema of the feet is present, nor during the prevalence of much debility—in fact, it should not, at any time, be carried farther, than just to produce the intended effect, as there are many other auxiliary arts in reserve.

"If the patient should be a strong woman, and if the disease has not been of long duration, twelve or fourteen ounces of blood may be taken away: if she should possess less strength of constitution, it may be sufficient to order the removal of six or eight ounces only: and to repeat this once in three weeks or a month. The application of leeches is to be very little depended upon." 93.

Has Mr. Clarke employed leeches to the pudendum muliebre in these cases? We are inclined to believe that they will be found, in most uterine affections, more serviceable there applied, than either cupping or leeching elsewhere.

All general and local stimuli are of course to be avoided—the diet to be of the mildest kind, as puddings, white fish, and vegetables. Wine and sexual connexion, to be entirely proscribed. The bowels should be so managed, that one easy motion be daily procured. All straining efforts in evacuating the rectum are highly injurious in uterine complaints, but especially in cauliflower excrescence. Fruit taken before breakfast, honey eaten instead of butter, a little manna eaten with a few blanched almonds, or a tea-spoonful of electuary of senna, will often obviate constipation. If these are not sufficient, the sulphate of magnesia in infusion of roses, will be found a mild laxative.

"The enlargement of the tumour may be greatly diminished, and the discharge consequently lessened, by the application of cold to the outside of the pelvis, and by the injection of cold fluids into the cavity of the vagina. Cold water may be applied to the external parts of generation, to the pubis, and to the loins, by means of a sponge; and this may be done, not once or twice only in the twenty-four hours, but several times: by keeping the parts in this way constantly chilled, the blood-vessels will be contracted, and the advantages resulting from such a mode of treatment will soon be made evident, in the diminution of the quantity of the discharge, and in the improvement of the constitutional health." 94.

The recumbent posture ought to be insisted upon. In injecting fluids, care should be taken that the syringe does not touch the excrescence, otherwise blood will flow. "A cylindrical syringe, the diameter of which is about three quarters of an inch, the extremity being rounded off, may be used for this purpose, and the patient should be cautioned not to introduce it farther than an inch, or an inch and a half."

In that aggravated form of the disease, where the tumour nearly protrudes, the patient should lie down upon the bed with her hips raised, and a small quantity of the astringent fluid should be poured in between the labia, with a common butter-boat. When the tumour has actually protruded, compresses dipped in an astringent fluid, may be applied, or a sponge wetted with it, may be lightly drawn over the surface. The astringent injections recommended, consist of sulphate of zinc and water, in various proportions, or alum, in a similar vehicle. Solutions of the mineral astringents in decoctions of astringent vegetables, constitute applications possessed of great power: such as, cort. granat. contus. ℥ss. aq. distillat. ℥xiij. coque per sextam partem horæ et cola, dein adde liquori colato aluminis ʒij. Galls, or oak-bark may be substituted for the pomegranate.

"The efficacy of the latter formulæ in a great measure depends upon the tannin. As this principle has the power of coagulating albumen, so as to form an insoluble precipitate, it becomes necessary to prepare the patient for a circumstance which may otherwise occasion great alarm in her mind,—the appearance of thin, whitish, or ash-coloured flakes which will come away from time to time. These are frequently thought to be portions of the body, and the agitation of the patient's mind has been very considerable, until it has been quieted by some explanation." 101.

Where irritable vagina exists, a mixture of decoction of oak-bark and linseed tea, forms a less irritating lotion.

In many cases, where the constitution has suffered, the powers of nature require to be recruited, and we must employ some light tonic. The muriatic and sulphuric acids are appropriate medicines. Sulphate of zinc, in such doses as do not excite vomiting, and combined with an essential oil to reconcile the stomach to its use, is recommended as occasionally useful:—say as follows:—sulphate of zinc, gr. xv. extract of hop, ʒj. oil of cinnamon, gt. iij. ♏. in pil. xv. one to be taken every night.

"The author is justified in repeating, that by a strict attention to and compliance with the rules above suggested, nearly every case of this disease may be made more tolerable; and, perhaps, such a change wrought in the size or the actions of the excrescence, in a few instances, as to remove all the symptoms." 104.

In some instances the resources of the medical art fail, and then the ligature holds out a prospect of relief, which has now and then been realized. True it is, that the fungus may, and probably will, be regenerated;—but a considerable time may elapse before a tumour of large size forms, and in the interim, by the removal of the secreting surface, the discharge will be restrained, and time will be afforded for the powers of the patient to recruit.

*Hydatids of the Uterus.* These are connected with the uterus and with each other by small filaments and by portions of substances partly bloody and partly gelatinous. A similar substance is attached to the internal part of the uterus, from which the footstalks of the hydatids grow. The number of these hydatids increasing, the cavity of the uterus enlarges, and when the organ has attained a large size, it seems to be offended by its contents, and then contracts upon them. The causes of this complaint are quite conjectural, or rather they are totally unknown.

When the pelvis can no longer contain the enlarged uterus, that viscus rises into the cavity of the abdomen, and may be felt as a circumscribed tumour through the parietes. The function of menstruation is usually interrupted.

"In the examination of a patient labouring under hydatids of the uterus, the body of this viscus will be found enlarged, and suddenly bulging out from the upper part of the cervix. All these symptoms attend other enlarged states of the uterus; but there remains to be mentioned one other symptom which serves to distinguish this disease from all others, and from pregnancy,—and this symptom is the discharge of an almost colourless watery fluid. This watery discharge is to be distinguished from that which attends the cauliflower-excrescence, by the irregularity and suddenness of its appearance and cessation; being produced by a rupture of one or more of the coats of these hydatids, in consequence of the occasional contraction of the uterus upon them, or of any sudden violence, as in the act of coughing or sneezing; whereas the discharge from the cauliflower-excrescence being a secretion from its surface is constantly escaping. The fluid watery discharge may be distinguished from those splashes of urine which sometimes come away from pregnant women, by being wholly inodorous." 118.

Sooner or later a parturient nisus takes place—the os uteri opens—the hydatids are expelled by periodical pains—and then, for the first time, danger presents itself in the form of a frightful hæmorrhage. The reason of this last is obvious. The placenta covers only a limited space of the internal surface of the uterus, whereas the hydatids spring from every portion of the cavity.

No means of curing or arresting the progress of this disease have hitherto been discovered. The patient is to be apprised of the nature of the complaint, and the event is to be patiently waited for, treating occasional symptoms as they arise. When the time arrives at which the uterus struggles to unload itself of its contents, then all the skill and energy of the practitioner will be necessary to control the hæmorrhage and sustain the powers of the constitution. Perfect quietude in the horizontal posture should be enjoined, and all stimulating food and drink denied. Cold applications are to be applied to the loins, abdomen, and external organs, and portions of ice, (their acute edges being rounded off by being held in the hand,) may be introduced into the vagina, or into the uterus.

"Let it not, however, be forgotten, that the great remedy for uterine hemorrhage is uterine contraction, and every possible mode of exciting this is to be put in practice. The application of a bandage round the abdomen has sometimes the power of exciting this contraction; but if the hemorrhage should continue profuse, and if any portion of the hydatids should remain in the uterus, an attempt should be made to remove these, in order to produce complete contraction of the muscular fibres." P. 121.

Two or three fingers, or the whole hand, should be covered with pomatum, carefully introduced into the uterus, and carried up between the sides of the uterus and the hydatids, which are to be detached from the part to which they adhere by the most gentle means.

"The mass, being now included in the hand of the operator, is to be brought out of the uterus, the surgeon recollecting always, in the performance of this operation, that the degree to which the os uteri is dilatable without laceration, is in proportion to the size of the whole uterus, both in pregnancy, as well as in this disease. So that, supposing the uterus in this disease to be enlarged to the size of that viscus in the sixth or seventh month of pregnancy, the whole hand of the operator may be, if necessary, introduced through the cervix; whereas, in smaller dimensions of the uterus, if any attempt is made to introduce the whole hand through the cervix, however carefully it may be attempted, a laceration of it may ensue, and thus the patient may be involved in a new danger." P. 122.

The expulsion over, and hæmorrhage restrained, the constitution must be invigorated by suitable means—particularly by the cinchona and mineral acids.

Another variety of this consists in a single cyst which distends the uterus. Our author has never met with a case of this kind. We need not therefore do more than refer to the usual sources of information on this subject. In the 34th and 35th sections of Burn's *fifth* edition, the reader will find information and references, on single and conglomerated hydatids.

*Oozing Tumour of the Labium.* In this, the discharge arises from the surface, or rather from the interstices of the tumour. The fluid is of a watery nature, and sometimes very abundant in quantity, being renewed almost immediately after the surface has been dried by a napkin. Blood never issues from the tumour, so that it has no analogy with cauliflower excrescence. The tumour is sometimes so large as to occupy the whole of the labia, extending even to the mons veneris. It seldom projects more than a line or two above the plane of the surrounding skin. The colour of the tumour varies little from that of the cuticle of the neighbouring parts.

"In the immediate neighbourhood of the tumour œdema is occasionally met with, but the tumour itself is not œdematous; soon after the surface of the tumour has been wiped quite dry, a watery fluid begins to ooze from it, and to form drops, which, having become large, at length run off, and keep the surrounding parts in a state of constant humidity; sometimes soreness and excoriation take place, as upon the upper lip, when the secretion from the nostrils is increased, but the tumour itself is seldom rendered more sensible." P. 129.

The secretion from this tumour corresponds, in appearance, with that from the cauliflower excrescence. The disease having begun, continues to enlarge, and insulated patches of it appear in the neighbouring parts, at length running into each other. Within the author's knowledge the complaint does not attack young women. The principal inconveniences of this disease are, an itching, sometimes preternatural sense of heat, and a watery discharge.

"When excoriations of the neighbouring parts are present, or an erysipelatous blush appears upon them, more advantage will be derived from the internal exhibition of the cinchona in substance, than from any other medicine; but no impression will be made upon the disease itself by this valuable remedy, and even the symptoms above mentioned will frequently recur, and call for the employment of the same remedies." 133.

A nutritious diet, and a moderate allowance of wine should be prescribed. External applications may mitigate but never cure the complaint. Common starch powder repeatedly sprinkled over the parts till it cakes upon them, is a very efficient remedy; but it will be necessary to keep the patient in the horizontal posture during its use—a position indeed which has a beneficial influence in itself.

"A mixture of starch-powder and cupri sulphas, very finely levigated, has been found serviceable; or a solution of cupri sulphas, or of argentum nitratum, may be used. A solution of gum arabic in decoctum quercûs may be tried. Cold water is also a valuable remedy, and there are no cases in which it will not afford much temporary comfort." P. 135.

Perhaps the most effectual applications are of a spirituous nature. Strong new port wine has afforded great relief; and when this has failed, brandy or arquebusade may be employed, or even alcohol. The complaint, upon the whole, is very rare.

*Involuntary Discharge of Urine.* Discharges of the urinary secretion, whether occasional or permanent, are troublesome, and often ulcerate the parts over which they pass. At the close of pregnancy, and in other enlargements of the uterus, the bladder can contain but small quantities of water, and this is often squeezed out in the act of coughing, laughing, or straining. For this case there is no remedy. Flat sponges sewed into the folds of a napkin should be constantly worn. If the inability depend on local or general debility, applications of cold water, bark, and the mineral acids, &c. may be used. If these fail, the lytta may be cautiously tried, or electricity. When all these are unsuccessful, mechanical means of compressing the meatus urinarius must be employed.

When a communication exists between the posterior part of the neck of the bladder and the anterior portion of the vagina, a most distressing case of involuntary discharge of urine will be the result.

"When the mischief has arisen from laceration or sloughing, it may be worth while to introduce into the vagina a large thin globular pessary, made either of wood or (which is better) of silver, perforated by a great number of holes, capable of containing a large piece of sponge. At the lower part of this pessary there should be a circular opening, through which sponges may be removed occasionally; and for this purpose a piece of string may be attached to the sponge, which, being emptied of the urine contained in it, may be again introduced into the cavity of the pessary, without the removal of it from the vagina." P. 144.

The above extract has brought us to the *third* and last chapter of the work, which, however, occupies nearly half the volume. It embraces the important class of *purulent* discharges, and will furnish a short analytical article for our succeeding number.

Of what use would it be to offer an opinion of a work, of which we have presented so copious an analysis? Many hundreds of our readers can appreciate the value of Mr. Clarke's practical precepts far better than we can. The wide experience, the known talents, and the unquestionable accuracy of Mr. Clarke, stamp a great value upon any work emanating from such a source; and we are happy in having the opportunity of selecting a part of our literary freight, this quarter, from a granary of such rich and precious materials. These materials are now sailing on the four winds, consigned to multitudes of unknown purchasers. We wish them a prosperous voyage, and bespeak for them a kindly reception.

Sive per syrtes iter æstuosas,
Sive facturus per inhospitalem
Caucasum, vel quæ loca fabulosus
Lambit Hydaspes.

